# A Pilot Study to Evaluate Haemostatic Function, following Shock Wave Lithotripsy (SWL) for the Treatment of Solitary Kidney Stones

**DOI:** 10.1371/journal.pone.0125840

**Published:** 2015-05-04

**Authors:** Stephen Fôn Hughes, Samantha Jayne Thomas-Wright, Joseph Banwell, Rachel Williams, Alyson Jayne Moyes, Sohail Mushtaq, Mohamed Abdulmajed, Iqbal Shergill

**Affiliations:** 1 Department of Biological Sciences, University of Chester, Chester, United Kingdom; 2 North Wales & North West Urological Research Centre (NW2URC), University of Chester, Chester, United Kingdom; 3 Haematology Department, BCUHB Wrexham Maelor Hospital, Wrexham, North Wales, United Kingdom; 4 Clinical Biochemistry Department, BCUHB Wrexham Maelor Hospital, Wrexham, North Wales, United Kingdom; 5 Department of Clinical Sciences & Nutrition, University of Chester, Chester, United Kingdom; 6 Department of Urology, BCUHB Wrexham Maelor Hospital, Wrexham, North Wales, United Kingdom; Rutgers University, UNITED STATES

## Abstract

**Purpose:**

The number of patients undergoing shock wave lithotripsy (SWL) in the UK for solitary unilateral kidney stones is increasing annually. The development of postoperative complications such as haematuria and sepsis following SWL is likely to increase. Comparing a range of biological markers with the aim of monitoring or predicting postoperative complications following SWL has not been extensively researched. The main purpose of this pilot-study was to test the hypothesis that SWL results in changes to haemostatic function. Subsequently, this pilot-study would form a sound basis to undertake future investigations involving larger cohorts.

**Methods:**

Twelve patients undergoing SWL for solitary unilateral kidney stones were recruited. From patients (8 male and 4 females) aged between 31–72 years (median—43 years), venous blood samples were collected pre-operatively (baseline), at 30, 120 and 240 minutes postoperatively. Specific haemostatic biomarkers [platelet counts, prothrombin time (PT), activated partial thromboplastin time (aPTT), fibrinogen, D-dimer, von Willebrand Factor (vWF), sE-selectin and plasma viscosity (PV)] were measured.

**Results:**

Platelet counts and fibrinogen concentration were significantly decreased following SWL (p = 0.027 and p = 0.014 respectively), while D-dimer and vWF levels significantly increased following SWL (p = 0.019 and p = 0.001 respectively). PT, APTT, sE-selectin and PV parameters were not significantly changed following SWL (p>0.05).

**Conclusions:**

Changes to specific biomarkers such as plasma fibrinogen and vWF suggest that these represent a more clinically relevant assessment of the extent of haemostatic involvement following SWL. Analysis of such markers, in the future, may potentially provide valuable data on “normal” response after lithotripsy, and could be expanded to identify or predict those patients at risk of coagulopathy following SWL. The validation and reliability will be assessed through the assessment of larger cohorts.

## Introduction

The prevalence and incidence of kidney stones is increasing worldwide [1]. Treatment methods for kidney stones have significantly improved with the introduction of shock wave lithotripsy (SWL), and this relatively non-invasive form of treatment has replaced open surgical procedures for the treatment of many kidney stones [2]. More recently, it has been noted that the number of SWL treatments for solitary unilateral kidney stones have been increasing annually, associated with a 63% rise in upper urinary tract stones being reported in the UK over a 10-year period [3].

Whilst SWL is an efficient treatment for urinary stones, numerous reports of haemostatic associated complications such as haematuria, as well as potentially critical intraparenchymal haemorrhage, subcapsular haematoma, and perirenal bleeding have been reported [4–5]. In addition, there is evidence to suggest that even brief exposure to shock waves may induce changes to the renal microvasculature, thus increasing the risk of such complications [6]. Furthermore, recent evidence also suggests that bleeding may initiate an inflammatory response that could result in scarring with permanent loss of functional renal volume [7]. As such, understanding the role of haemostatic parameters may be beneficial in monitoring or predicting postoperative complications after treatments such as SWL.

Disturbances to the normal vascular integrity, due to SWL, may result in abnormal haemostasis, which can lead to bleeding or thromboembolic complications. Platelets play a critical role during primary haemostasis (platelet plug formation) and aid in the development of blood clotting. Although increased platelet counts have been reported to be dominant contributors to hypercoagulability after injury in surgical intensive care trauma patients, little evidence has been documented with respect to changes in platelet concentrations following SWL [8]. With regards to secondary haemostasis (coagulation), prothrombin time (PT) and activated partial thromboplastin time (aPTT) are performance indicator tests that measure the extrinsic and intrinsic coagulation pathways. A recent study by Dedej *et al* (2013) has demonstrated alterations in haemostasis following abdominal surgery, where PT decreased significantly from 90.38% preoperative to 81.25% at 72 hours postoperative [9]. Fibrinogen is a large soluble and complex glycoprotein that is converted by thrombin into fibrin during blood clot formation. Fibrin degradation products are formed whenever fibrin is broken down by enzymes (for example, plasmin). D-dimer is an end product derived from plasmin-mediated degradation of cross-linked fibrin clots. D-dimer measurement has proved to be a sensitive marker for the evaluation of disseminated intravascular coagulation (DIC) [10]. von Willebrand Factor (vWF) is a large multimeric glycoprotein and performs essential functions of haemostasis *in vivo*, through its variety of bridging and binding activities. Specific functions of vWF include its ability to act as an essential co-factor for factor VIII in the blood coagulation cascade, platelet adhesion, platelet aggregation and platelet plug stabilisation. vWF has been reported as being an established marker of endothelial activation [11–13].

E-selectin (CD62) is a cell adhesion molecule expressed on endothelial cells, which is activated by cytokines and facilitates leukocyte-endothelial interactions. Soluble E-selectin (sE-selectin) is found in the blood, probably arising from proteolytic cleavage of the surface-expressed molecule [14]. Elevated levels of E-selectin have been reported in patients with sepsis [15]. Plasma viscosity provides a simple and reliable way of monitoring changes in plasma proteins as a result of trauma, inflammation or infection. During an inflammatory process, it can be appreciated that the high proportion of fibrinogen in the blood causes red blood cells to stick to each other, thus increasing plasma viscosity values.

Whilst we, and others, have previously reported post-operative changes in haemostatic markers following various surgical procedures [13, 9, 16], crucially, there is very little evidence investigating the direct effect of SWL on haemostatic parameters. Hence, the aim of this pilot study, was to test the hypothesis that SWL results in changes to haemostatic function, increasing endothelial and haemostatic involvement postoperatively. We aimed to evaluate changes in selective haemostatic and endothelial biomarkers, to provide a better understanding of their postoperative course following elective SWL, for the treatment of solitary unilateral kidney stones. This pilot-study would provide a sound platform to undertake future investigations involving larger cohorts of SWL patients.

## Methods

### Subject Volunteers and Shock Wave Lithotripsy (SWL)

Ethical approval for this pilot study was received from the Wales Research Ethics Service (REC) 4 committee (REC4: 12/WA/0117). Twelve consecutive patients scheduled for elective SWL for solitary unilateral kidney stones, were recruited after providing written informed consent to participate in the study (n = 12). The patients (8 males and 4 females) were aged between 31 and 72 years old (median age = 43 years). Each patient served as their own control as data was taken from baseline values (pre-treatment) through the various time points post-treatment. Any changes in the haemostatic markers measured were due to the non-invasive procedure as these patients did not undergo anaesthesia (general or local). Treatment was given as per standardised protocol using Wolf P3000 device, incorporating triple focus technology, with ultrasound and fluoroscopic imaging as appropriate.

### Blood Samples

Prior to SWL, a cannula was inserted into the ante-cubital fossa, and a venous blood sample was collected pre-operatively, which stood as a baseline measurement for that particular patient. After SWL, further blood samples were collected at 30 minutes, 120 minutes and 240 minutes post-operatively. The blood samples were collected into vacutainers containing di-potassium ethylene diamine tetra-aceticacid (EDTA) and tri-sodium citrate. Subject plasma was obtained by centrifuging whole blood samples at 450g for 15 minutes, following which all plasma samples were stored (-80°C) until required for further analysis.

### Measurement of platelet concentration

Complete blood counts were performed using a Sysmex XE-5000 automated cell counter and platelets measured in units x10^9^/L.

### Measurement of prothrombin time (PT), activated partial thromboplastin time (aPTT) and fibrinogen

PT (seconds) was measured in citrated samples, using a Randox Monza semi-automated system as described by the manufacturer’s instructions (Randox RX Monza Method Sheet: PTH 2752). aPTT (seconds) and fibrinogen (g/L) were measured using citrated samples on a Sysmex CS2100 analyser.

### Measurement of D-dimer, vWF and sE-selectin

D-dimer (ng/mL) was measured by a two-step enzyme immunoassay sandwich method, with a final fluorescent detection as described by others [17]. Measurement of this parameter was performed with a Mini-Vidas automated immunoassay system (Biomerieux, UK), using Enzyme-Linked Fluorescent Assay technology. Plasma vWF concentration (IU/dL) was measured as described previously by a sandwich-type ELISA technique, using rabbit anti-human vWF and rabbit anti-human vWF peroxidase conjugate (Dako, UK), [11, 18–19]. Measurement of sE-selectin (ng/mL) was performed using commercially available kits supplied by R&D Systems Europe, and involved using ELISA assay as described by the manufacturer (R&D Systems, Catalogue # SSLE00).

### Measurement of plasma viscosity (PV)

Plasma viscosity (mPa/second) was measured using a Benson BV200 viscometer.

### Statistical Analysis

All results were presented as mean ± standard error (SE) or median ± Iqr. Where data was normally distributed, repeated measures one-way analysis of variance (ANOVA) between samples test was employed, adopting a 5% level of significance. Post hoc testing was conducted using the Bonferroni test for pairwise comparisons between means. Data that did not comply with normality were analysed using the Friedman test. Where the Friedman test resulted in statistical significance, subsequent tests were performed using the Wilcoxon test. Statistical significance was accepted when p ≤ 0.05.

## Results

### Platelet Count

**Fig 1:**
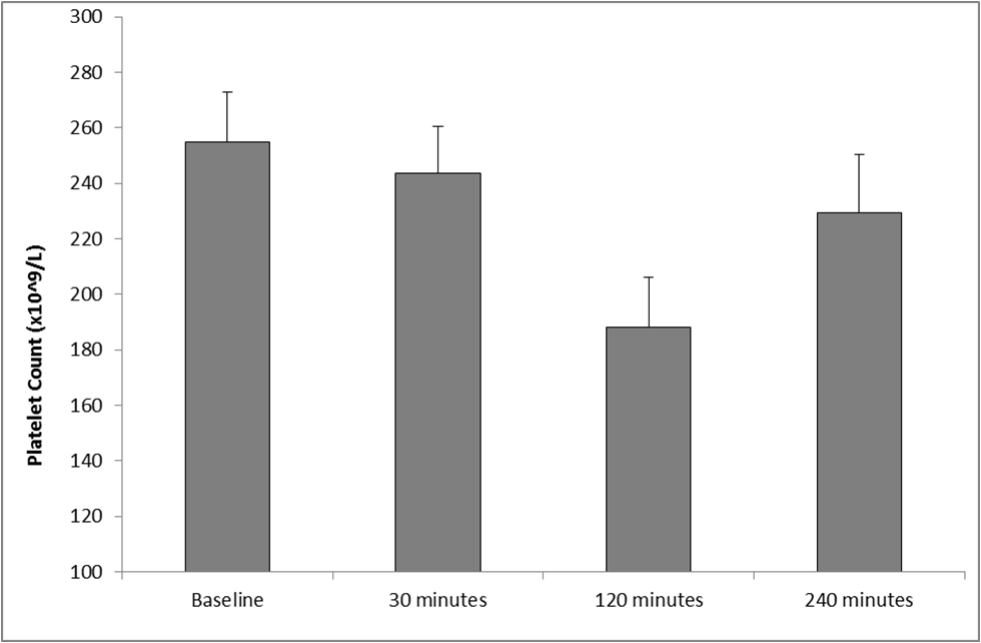
Effect of SWL, for the treatment of kidney stones, on platelet count (n = 12). The points represent mean ± SE, p = 0.027 as determined ANOVA.

As displayed in Fig 1, following SWL, significant changes, compared to baseline, in platelet concentration were observed (p = 0.027, as determined by ANOVA). Specifically, platelet values decreased from baseline (255.08 ± 19.97), during 30 minutes (243.67 ± 17.28) and 120 minutes postoperative (188.33 ± 18.21). At 240 minutes postoperative platelet counts increased (229.56 ± 20.58), although remained lower to those of basal values. Upon further analysis, pairwise comparisons showed no significant differences between baseline *vs* 30, 120 and 240 minutes postoperative (p>0.05).

### Fibrinogen

**Fig 2:**
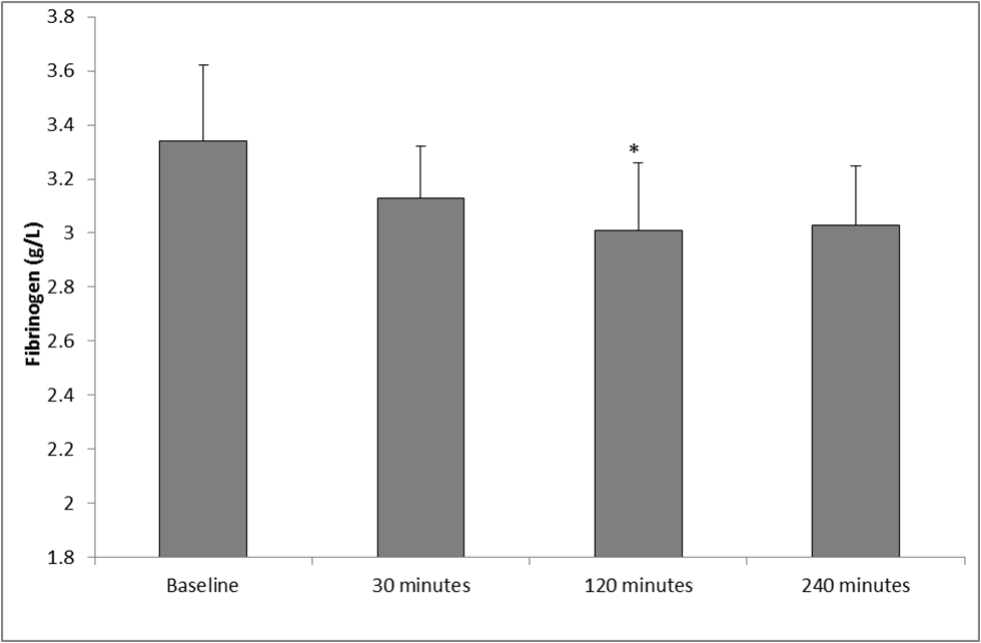
Effect of SWL, for the treatment of kidney stones, on fibrinogen concentration (n = 12). The points represent mean ± SE, p = 0.014 as determined by the ANOVA test. *p = 0.026 baseline (preoperative) *vs* 120 minutes postoperative as determined by the Bonferroni test for pairwise comparisons.

Fig 2 shows the changes in fibrinogen concentration following SWL. Following SWL, a significant decrease in fibrinogen concentration was observed (p = 0.014, as determined by ANOVA). Fibrinogen levels decreased from baseline (3.34 ± 0.28), during 30 and 120 minutes postoperative (3.13 ± 0.19, 3.01 ± 0.25 respectively). Fibrinogen concentration increased at 240 minutes postoperative (3.03 ± 0.22), although remained lower to those of basal values. Upon further analysis, pairwise comparisons showed significant differences between baseline (pre-operative) *vs* 120 minutes postoperative (p = 0.026).

### Von Willebrand Factor

**Fig 3:**
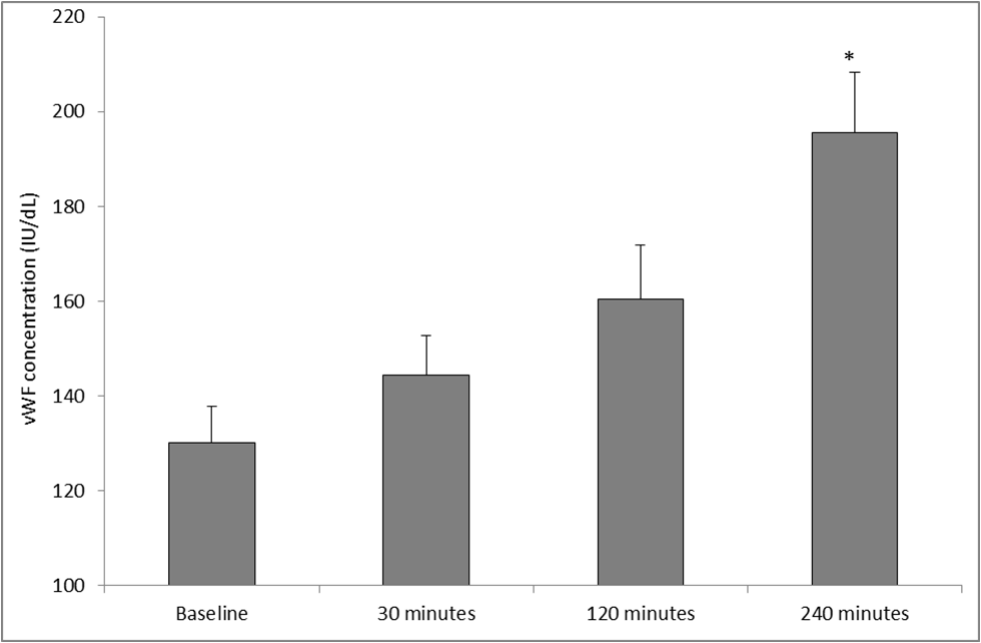
Effect of SWL, for the treatment of kidney stones, on vWF concentration (n = 12). The points represent mean ± SE, p = 0.001 as determined by ANOVA. *p = 0.005 baseline (preoperative) *vs* 240 minutes postoperative, as determined by the Bonferroni test for pairwise comparisons.

The results for changes in vWF are shown in Fig 3. Following SWL, a significant increase in vWF was observed (p = 0.001, as determined by ANOVA). vWF increased from baseline (130.02 ± 7.72), during 30 minutes (144.30 ± 8.36), 120 minutes (160.39 ± 11.46), and peaking at 240 minutes (195.51 ± 12.74) postoperative. Upon further analysis, pairwise comparisons showed significant differences between baseline (preoperative) *vs* 240 minutes postoperative (p = 0.005).

### Prothrombin time (PT) and activated partial thromboplastin time (aPTT)

Changes in PT and aPTT following SWL are described in Table 1. As can be observed, PT increased from baseline (12.03 ± 0.43), at 30 minutes post-operatively (13.08 ± 0.66) and peaking at 120 minutes (13.47 ± 0.89) postoperative. PT decreased toward basal levels at 240 minutes (13.14 ± 0.57) postoperative, although remained at a higher level to those of basal values. With respect to the aPTT, there were no changes from baseline (24 ± 23) and 240 minutes postoperative (24 ± 23). However, following SWL, no significant changes were observed in PT and aPTT (p = 0.139 and p = 0.464 respectively).

**Table 1:** Effect of SWL, for the treatment of kidney stones, on PT, aPPT, D-dimer, sE-Selectin and PV, (n = 12).

	Baseline	30 minutes	120 minutes	240 minutes	p value	Statistical Test
**PT (s)**	12.03 ± 0.43	13.08 ± 0.66	13.47 ± 0.89	13.14 ± 0.57	0.139	ANOVA (± SE)
**aPPT (s)**	24 ± 23	24 ± 23	24 ± 23	24 ± 23	0.464	Friedman (± Iqr)
**D-Dimer (ng/ml)**	245 ± 168	429 ± 252	1233 ± 323*	568 ± 357*	0.019	Friedman (± Iqr)
**sE-selectin (ng/ml)**	84 ± 40	84 ± 46	66.8 ± 41	66.2 ± 39	0.269	Friedman (± Iqr)
**PV (mPa/s)**	1.66 ± 0.03	1.64 ± 0.04	1.56 ± 0.03	1.55 ± 0.04	0.129	ANOVA (± SE)

*p<0.05 (D-dimer): Baseline (preoperative) vs 120 (p = 0.008) and 240 (p = 0.038) minutes postoperative, as determined by the Wilcoxon test.

### D-dimer

Following SWL, a significant increase in D-dimer was observed, p = 0.019, as determined by the Friedman test (Table 1). Specifically, D-dimer increased from baseline (245 ± 168), during 30 minutes (429 ± 252) and peaking at 120 minutes (1233 ± 323) postoperative. D-dimer decreased toward basal levels at 240 minutes (568 ± 357) postoperative, although remained at a higher level (2 fold) to those of basal values. Upon further analysis the Wilcoxon test showed significant differences between baseline (preoperative) *vs* 120 and 24 0 minutes postoperative (p = 0.008 and p = 0.038 respectively).

### sE-selectin

There was no change in sE-selectin values (Table 1) from baseline (84 ± 40) up to 30 minutes postoperative (84 ± 46), although trends of decreasing concentrations was observed at 120 and 240 minutes postoperative (66.8 ± 41 and 66.2 ± 39 respectively). However, following SWL, no significant changes to sE-selectin was observed (p = 0.269, as determined by the Friedman test).

### Plasma Viscosity

Table 1 demonstrates the changes in plasma viscosity following SWL. PV decreased from baseline (1.66 ± 0.03 preoperative), during 30 (1.64 ± 0.04), 120 (1.56 ± 0.03) and 240 minutes postoperative (1.55 ± 0.04). However, there were no significant changes to PV following SWL (p = 0.129, as determined by ANOVA),

## Discussion

The main aim of this pilot-study was to evaluate changes in selective haemostatic and endothelial biomarkers, to provide a better understanding of their postoperative course following elective SWL, for the treatment of solitary unilateral kidney stones. As such, we have shown that significant changes in several haemostatic biomarkers do occur following SWL.

During the present study, none of the patients developed any complications in the immediate or follow up period, clinically or radiologically. As such, this pilot study provides potentially valuable data on “normal” response after lithotripsy. However, our study demonstrated a significant increase in vWF from baseline up to 240 minutes (4 hours) postoperatively. This is consistent with our previous reports of increasing vWF concentrations in the post-operative period following other surgical procedures [13]. The inference is that SWL may result in an increased liberation of vWF from the storage organelles of the vascular endothelium following treatment, causing disruption to vascular integrity, which may lead to bleeding or thromboembolic complications. Results from this study also showed decreasing platelet concentration following SWL, providing evidence of diminished thrombocytic activity during the postoperative period. Our findings compliment the work by Dedej (2013) who reported similar findings of decreased platelet values at 72 hours following abdominal surgery [9]. Clearly, if reproduced in subsequent studies, this finding may have possible clinical implications, in potentially identifying or predicting those patients at highest risk of developing haematuria or renal haematomas following SWL. A possible explanation for the changes of platelet counts and fibrinogen observed during this study might be the redistribution of them in the body due to renal trauma caused by SWL, by which the platelet counts and fibrinogen will be increased at the kidney (the traumatized organ) immediate after SWL, and decreased at the peripheral blood. Subsequently, after 2 or 3 hours these parameters may regain their normal distribution. Theoretically, this may enhance intrinsic haemostatic activity in the early post-SWL period.

Measurement of D-dimer, which is a marker of haemostatic activity, showed a significant increase in concentration following SWL (p = 0.019). These observations were similar to those of Umekawa *et al* (1993), who reported a transient acceleration in fibrin degradation products (FDP) and D-dimer levels following SWL [20]. Interestingly, no significant changes were observed in the PT and aPTT following SWL, although fibrinogen concentration decreased significantly (p = 0.014). Changes to fibrinogen levels are known to lead to either bleeding or thromboembolic complications. A recent study by Yang *et al* (2013) demonstrated that lower postoperative fibrinogen levels were associated with bleeding after cardiothoracic surgery [21]. Our finding followed a similar pattern of decreasing fibrinogen concentration up to 240 minutes (4 hours) following SWL, and requires further investigation to clarify the role of fibrinogen as a potentially modifiable risk factor for perioperative bleeding, that may occur following SWL. With regards to sE-selectin and plasma viscosity, no significant changes were observed in these parameters.

To date, there are very few reports on the effect of SWL, for the treatment of kidney stones, on haemostatic function, and as such, the results from this study are a welcome addition to the urological literature. We have shown that changes to specific biomarkers, such as fibrinogen and vWF concentrations, occur following SWL. If these results are reproduced in larger studies, there is a putative suggestion that these markers may provide a more clinically relevant assessment of the extent of haemostatic involvement following SWL.

It is acknowledged that the main limiting factor of this pilot-study is the relatively small number of patients recruited (n = 12), but we feel that further work can be performed to look at these haemostatic markers, as well as inflammatory markers and their correlation with clinical outcome (e.g. bleeding and infection), in a larger cohort. Although none of the patients during this study had an untoward outcome, it would be beneficial for future investigations to include a control other than the patients themselves. Although ethical approval would not be granted for SWL to be performed with normal healthy individuals (i.e. those without kidney stones); comparing patients undergoing flexible ureteroscopy, for the treatment of kidney stones, would provide an alternative control measure, and help generate unique results to establish the extent of inflammation and changes to haemostatic parameters following both procedures.

It would also have been interesting to have followed up the patients with regards to measurement of their biological markers at review clinic’s, this could have indicated any continued haemostatic reactions post-treatment, which may have had an impact in supporting surgeons with their management strategies of patients during the post-operative period.

Ultimately, if changes to haemostatic markers following SWL can identify or predict those patients at increased risk of coagulopathy, future directions of this study may offer the potential of pharmacological intervention. For example, using anti-haemorrhagic agents as preventative measures for post-operative bleeding, could improve patients’ recovery following SWL for the treatment of kidney stones.

In conclusion, changes to specific biomarkers such as plasma fibrinogen and vWF suggest that these represent a more clinically relevant assessment of the extent of haemostatic involvement following SWL. Analysis of such markers, in the future, may potentially provide valuable data on “normal” response after lithotripsy, and could be expanded to identify or predict those patients at risk of coagulopathy following SWL. The validation and reliability will be assessed through the assessment of larger cohorts.
